# Sticky Policies, Dysfunctional Systems: Path Dependency and the Problems of Government Funding for Science in the United States

**DOI:** 10.1007/s11024-020-09409-2

**Published:** 2020-06-11

**Authors:** Frank N. Laird

**Affiliations:** Josef Korbel School of International Studies, University of Denver, 2201 S. Gaylord St., Denver, CO 80208 USA

**Keywords:** Science policy, Government R&D funding, Historical analysis, Institutions, Path dependency

## Abstract

Leaders of the scientific community have declared that American science is in a crisis due to inadequate federal funding. They misconstrue the problem; its roots lie instead in the institutional interactions between federal funding agencies and higher education. After World War II, science policy elites advocated for a system of funding that addressed what they perceived at the time as their most pressing problems of science-government relations: the need for greater federal funding for science, especially to universities, while maintaining scientific autonomy in the distribution and use of those funds. The agencies that fund university research developed institutional rules, norms, and procedures that created unintended consequences when they interacted with those of American higher education. The project system for funding, justified by peer-review and coupled with rapidly increasing R&D budgets, created incentives for universities to expand their research programs massively, which led to unsustainable growth in the demand for federal research money. That system produced spectacular successes but also created the unintended longer-term problem that demand for science funding has grown more quickly than government funding ever could. Most analysts neglect potentially painful reforms that might address these problems. This case demonstrates that successful political coalitions can create intractable long-term problems for themselves.

## Introduction

Leaders of the scientific community have declared that American science is in decline and that the United States is losing its global scientific and engineering preeminence as other countries invest in improving theirs. The purported consequences of this decline will be nothing less than economic stagnation and a loss of national security. A 2007 National Academy of Sciences (NAS) study framed the problem with its title: *Rising Above the Gathering Storm*. The study concluded that if the United States does not fix the problem, then “[f]or the first time in generations, our children could face poorer prospects for jobs, healthcare, security, and overall standard of living than have their parents and grandparents” (p. 223). The NAS study identified many causes of the decline, from restrictions on immigration to K-12 education, but the heart of the matter was too little federal funding for research, especially for universities. What the NAS study did *not* recognize is that those funding problems had arisen from deeply embedded institutional features of federal science policy, not a stingy Congress. The fundamental problem is that *interactions* between federal agencies and higher education have led to unintended consequences, creating an induced demand for research and development (R&D) funding for universities that has increased more quickly than R&D budgets ever could. In turn, that imbalance has led to increasing pressure on scientists operating within that system (Bok 2015: 328–29; Howard and Laird 2013). Solving this problem requires challenging the core institutional features of the existing system. The historical development of that system reveals that the scientific community, especially in universities, has become dependent on a persistent but dysfunctional system (Pierson 2004: 18). That dysfunction arises from core features of the policy itself. Science policy elites have sought, without success, simply to push aside the constraints of budget policy-making instead of changing those core features.

## World War II’s Changes to U.S. Science Funding

The current American research funding system arose after World War II. The story is well-known (see Greenberg 1967; Hart 1998; H.R. Rpt. 1986a; Kleinman 1995), but I summarize it here to analyze some under-appreciated features of science policy that have led to the current problems with the system. Prior to the war, government-funded research was, in most fields, small, consistent with total federal spending, which was a small part of the economy (OMB 2018). While the New Deal greatly expanded the overall role of the federal government, federal R&D funding did not partake in that growth. Instead, it was the war that provided a critical juncture for science policy, a historical moment that made it possible to institute radically new policies, which set the stage for the creation of new peacetime institutional rules and norms that would “trigger a process of positive feedback” (Pierson 2004: 51, n. 26). Creating new rules and norms required challenging entrenched views within the scientific community, as well as within the federal government. Odd as it may seem today, before World War II many leaders of the scientific community resisted increased federal funding out of fear that government control would accompany such funding (Greenberg 1967: 58–61). Moreover, conservative Americans hostile to government programs in general included many people in the scientific and engineering community (Hart 1998, chap. 1).

As a result, the American scientific community devoted to basic research (“pure science” in an earlier idiom) was small and, in most fields of science, a backwater compared to that in Europe. In December 1926, then-Secretary of Commerce Herbert Hoover bemoaned the state of pure science in America, blaming it on the heavy emphasis government and industry put on applied science. He estimated that government and industry together spent about $200 million per year on applied science but that government and philanthropy together only spent about $10 million on pure science (Hoover 1927: 26–27). While Hoover was President, 1929–33, he raised funding for pure science to about $25 million, but during the New Deal it fell back to the $10 million range.^1^ During this era, American science faculty would often urge their best undergraduates to pursue PhDs in Europe (Greenberg 1967: 56–59). Private firms focused on their immediate technological needs and so spent little on basic research. The federal government did have a few agencies with substantial R&D programs, but much of that money went to government facilities or private firms in the relevant industries (e.g. Ruttan 2006, chap. 3). Only the Department of Agriculture had developed an extensive university-based research program (DuPree [1957] 1986: 182 and 332; H.R. Rep 1986a: 9–10).

During the 1930s, some leaders within the scientific community argued that scientists could benefit from greater government support *if* scientists themselves had control over how the money was spent. Karl Compton, then president of both the Massachusetts Institute Technology (MIT) and the American Institute of Physics, argued that greater investments in science would benefit science and the larger society (Compton 1934: 299). Compton compared government funding for research in the United States unfavorably with several other countries, including Russia and Japan, and concluded that “we have been more lucky than intelligent” (Compton 1935: 354). Abraham Flexner, director of the Institute for Advanced Study, argued in 1939 for both greatly increased government support of basic research and scientific autonomy in his pamphlet *The Usefulness of Useless Knowledge*. Great practical benefits, he claimed, came from scientists who had no interest in the applications of their ideas, but whose ideas then provided the basis for important new technologies (Flexner [1939] 2017), an early version of what became the linear model of technological innovation.

Science policy entrepreneurs successfully used the crisis of the war to end the old system of meager government funding for science. In June 1940, before the United States formally entered World War II, President Franklin D. Roosevelt created the National Defense Research Council, a year later expanded and renamed the Office of Scientific Research and Development (OSRD), to fund and coordinate war-related research. Roosevelt appointed Vannevar Bush, formerly of MIT, then president of the Carnegie Institution, as its director, making Bush the top White House official for science and technology (Baxter 1947, chap. 1 and Appendices 1 and 2; Zachary 1997, chaps. 7–10). Bush oversaw an increase in funding for war-related R&D, including to universities, as OSRD funding shot up from $6.4 million in FY1941 to $145.5 million in FY1944 (Baxter 1947: 125). By the end of the war, large contracts to universities had totaled $323.3 million (Baxter 1947: 456–7). Additional R&D funding came from the military directly.

University scientists, who had spent years scrimping by on private philanthropy, suddenly saw what massive federal spending could do. This flood of money changed universities’ relationship to the federal government and, though this funding was concentrated at a small number of universities, its effects spread much more widely. MIT and the California Institute of Technology (Cal Tech) received, between them, $200.4 million of that $323.3 million total. Nonetheless, scientists from many universities had the experience of working with generous federal funding because federal research administrators had quickly realized that some projects required large, centralized laboratories, but no single university had the staff to operate such labs. Therefore, OSRD officials persuaded many universities to release some of their scientists and engineers for the duration of the war to go work in large labs at a few key universities. By the end of the war, the Radiation Lab at MIT had staff from 69 different colleges and universities, was directed by a physicist from the University of Rochester, and had only one MIT faculty member on its steering committee (Baxter 1947: 20–22). The same was true at the large labs at Cal Tech and Harvard. In addition, many academic scientists took leave from their universities to work in the new national laboratories that the government had established to create nuclear weapons and similar projects. Thus scientists and engineers from across academia experienced expansive federal funding. As James Baxter, a senior official in OSRD, put it, “Money was never a limiting factor . . . As the agency grew, Congress proved unfailingly generous” (Baxter 1947: 19).

As the director of OSRD, Bush could fund any institution he wished using almost any contractual arrangement, and he experimented constantly with new procedures for distributing the money, in the process creating new institutional arrangements on the fly (Baxter 1947, chap. 1). These qualitative changes to federal science policy were just as important as the increasing budgets for laying the foundations for the post-war R&D funding system. New institutional rules determined which scientists or institutions would get federal money, who made those decisions, and based on what criteria. Those rules became the focus of protracted and heated debate even before the war ended. Starting in 1942, Senator Harley Kilgore (D-WV) introduced bills to create an agency for war-time and post-war R&D. Kilgore’s bills emphasized directing scientific research to national needs and having diverse constituencies involved in deciding which projects to fund. His bills drew strong opposition from the Army, the Navy, industrialists, scientific organizations, and, not least, from Vannevar Bush and his allies in and out of government. Bush and Kilgore differed fundamentally over key issues, especially about how much control scientists outside of government would exert over the distributions of federal R&D funds and toward what ends those funds would aim (Kevles 1977: 9–16). These conflicts reflected fundamentally different visions of the role of the state among American political elites (Hart 1998, chap. 1) and highlight how contingent the final result was.

## Building Post-War Institutions

By 1943 Bush quietly began planning for a new post-war system, convinced that the federal government should maintain substantial funding for scientists engaged in basic research. In 1944 he persuaded President Roosevelt to request a report on post-war science policy, which Bush and his staff then produced (Zachary 1997: 219–224 and endnote 15). *Science: The Endless Frontier,* released in 1945, became the Ur-text of American science policy, even though Bush did not get everything that he wanted in post-war science institutions. Though different government agencies adopted different methods for distributing R&D funds, the overall system implemented key components of Bush’s vision, namely, greatly increased funding for science while allowing the scientific community outside of government to exercise substantial control over the allocation of the funds. Bush, a skilled policy entrepreneur, framed his proposals in terms of the need for the United States to invest heavily in scientific research to maintain a strong military technological advantage, along with promoting prosperity and public health (Hart 1998: 24–25; Zachary 1997, chap. 10). During the war, Bush’s office had funded research that produced a remarkable array of technological innovations (Baxter 1947 catalogs them), which gave him considerable credibility with political elites thereafter. Nonetheless, despite Bush’s influence, it took five years after the end of the war for Congress to pass the legislation that created the National Science Foundation (NSF) due to intense political battles over the purpose and structure of post-war federal science funding, mainly with the allies of Senator Kilgore (H.R. Rep. 1986a; Hart 1998; Zachary 1997). The final form of federal R&D funding, spread across multiple government agencies, was a hybrid system that resulted from a set of political compromises. Some agencies adopted Bush’s ideas, while others used older systems of mission-oriented funding.

The National Institutes of Health (NIH), the newly-created NSF, and portions of other agencies emphasized funding mostly untargeted basic research. But this system is not the only way to fund science, as other countries and other U.S. government agencies, such as the Departments of Agriculture and Defense, have demonstrated (H.R. Rep. 1986b; Dupree [1957] 1986, chap. 8). To justify such public funding, Bush and his allies needed to explain how untargeted research created tangible benefits for the country. Absent such benefits, why should the government fund basic science at levels higher than those it provided for, say, the arts and humanities? The linear model of technological innovation, articulated in the 1930s by Flexner ((1939) 2017) and others, provided that justification. That model stated that the country needed to support basic research as the “seed corn” that would give rise to new technologies. This model assumes that private firms will underinvest in basic research because its benefits are uncertain and long-term and because the firms cannot capture all the benefits of their investment. Due to this classic example of a public goods market failure, the federal government must step in to provide that investment (Brooks 1990: 169–170; Nelson 2005: 225–230). Though Bush himself knew that the linear model was far too simple to explain scientific and technological advances, he understood that it was a politically expedient way to justify government support for basic science, and *Science: The Endless Frontier* embraced this model (Zachary 1997: 219; H.R. Rep 1986a: 22–23).

Proponents of the linear model claimed that government agencies should not try to pick the most “practical” basic research topics, since no one can predict which basic scientific results will lead to the most or most important technological applications. Instead, the government should fund the “best” basic science. To operationalize this model of choosing the best science, agencies should fund individual projects, as opposed to providing steady funding to particular research programs or universities. This apparently mundane administrative procedure—providing funds to individual projects instead of particular people, programs, universities, or even geographic regions—became the truly disruptive institutional feature of post-war science policy. That a scientist worked at a prestigious university or had previously received government funding was no guarantee that he or she would continue to produce the best science, and funding by geographic regions or some other formula could deprive more deserving projects of funding if they fell outside the bounds of that formula. The only way to insure that money went to the best science was to subject every scientific effort, project by project, to rigorous review. No scientist or university could rest on its laurels or geographic good luck.

Not all federal funding agencies chose the project funding system, but those that did used peer review by scientists outside of government as their method for choosing the best projects to fund (Polanyi 1962 for the classic justification of this procedure). Whatever the strengths and weaknesses of peer review (Smith 1990: 179–184 for a review), it institutionalized the norm that the non-governmental community of scientists should have a major say over the distribution of research funds and justified autonomy for scientists who are conducting research (Chubin and Hackett 1990: 9, 19–20), making it part of the “social contract for science” (Guston and Keniston 1994: 1–2). Unlike any other government distributional policy, science policy places much of the control of that distribution in the hands of those who, as a group, benefit from it. Congress determines the budgets of the various agencies and so implicitly allocates funds among broad fields of science, but the peer-reviewed project system ensures that non-governmental scientists greatly influence who actually receives the funds.^2^ The policy advocates who promoted this system believed that it would convince scientists that government funding could come without intrusive government control. In terms of gaining acceptance among policymakers and the wider public, peer review justified the competitive project system, claiming that it removed bias from the system and promoted only the most meritorious projects. The competitive project system, in turn, created the institutional incentives that led to an imbalance in supply and demand for R&D funds.

Moreover, this system of funding science fit neatly into the ideological struggle of the Cold War in that it rewarded individual excellence, independent of whoever happened to be in power in government at any given time (Wolfe 2018). The individual, competitive project grant became the dominant, though not only, funding mechanism the federal agencies have used to fund university research (H.R. Rep. 1986b, chapters 1 and 2).

The project system of funding is now so deeply ingrained at American universities, so taken for granted, that it seems utterly natural, yet, coupled with greatly increased funding after World War II, it radically changed the opportunities that universities faced. If federal funding could, in principle, go anywhere, and if that funding was increasing rapidly, then universities that had small research profiles could greatly increase their R&D activity and gain increased budgets, graduate programs, and prestige if they could successfully compete for such funding. Also, because status accrued to universities that had high-profile research and graduate programs, many universities wanted to enter those ranks. As a result, since the end of the war, more universities have required their faculty to compete successfully for grants and publish their research in order to get hired and promoted (Bok 2015: 328–29), thereby increasing the demand for research funds. This dynamic, a form of induced demand (Ladd 2012), planted the seeds of the current problems that science policy elites are labelling a crisis of funding.

These new policies and institutional practices succeeded for decades after the war, thanks to dramatically increased federal funding for R&D, spurred in part by the security pressures of the Cold War. Competition with the Soviet Union ran along many dimensions, including scientific and technological supremacy. Even agencies whose research had no military applications enjoyed growing budgets. The NIH budget increased by more than a factor of 10 in short order, shooting up from less than $3 million in 1945 to more than $52 million in 1950 (H.R. Rep. 1986a: 30). Between 1949 and 1957, total defense R&D rose by a factor of 2.4 in constant dollars, and total non-defense funding increased by a factor of 2.7 in the same period. (OMB 2018, Table 9.7). Yet even those rapid increases, almost tripling in real terms in less than ten years, looked meager compared to R&D budget growth after the Soviet Union launched Sputnik on October 4, 1957. Though President Dwight D. Eisenhower was not particularly concerned about Sputnik, other politicians and many segments of the media pushed the idea that the satellite demonstrated Soviet technological superiority and posed a major threat to national security (“Sputnik” 2006). In response, Congress accelerated R&D funding increases. Between fiscal years 1957 and 1967, defense R&D outlays went up by a factor of 3.2 in constant dollars, and real non-defense R&D spending shot up by a factor of 11.5. Even if one subtracts the space budget from the non-defense R&D total, the civilian R&D budget increased by a factor of 4.6 in real terms in that decade (OMB 2018, Table 9.8). By 1968 total R&D funding reached $16.17 billion in nominal terms, more than quintupling in a decade. During the same period the overall federal budget did not increase dramatically, instead remaining a few percentage points above or below 20% of GDP (OMB 2018, Table 1.2). Federal spending on R&D increased from 3.5% of federal outlays to 11.7% (OMB 2018, Table 9.7). It was a remarkable time to be a scientist in the United States. For more than 20 years after World War II, both scientists and science policy-makers could well believe that the American system for funding science was working splendidly.

Indeed, that system produced spectacular results. Researchers in the United States came to dominate the international scientific community. Since 1945 scientists working in the United States have won more than half of all the chemistry, physics, and medicine/physiology Nobel Prizes.^3^ Although researchers in the European Union and China both currently produce more papers than those in the United States, publications by Americans dominate the measure of the most cited papers (National Science Foundation 2018a, pp. O-17, O-22). In the Shanghai rankings of universities, 16 of the top 20 universities are American.^4^ Tens of thousands of scientists and engineers from around the world seek to come to the United States for graduate school or faculty positions (National Science Foundation 2018a, pp. 3–125, 3–126 and 5–53, 5–57). How could this system be a problem?

## Slowly-Building Problems

Universities responded to the incentives and opportunities that post-war science policy offered. The number of U.S. universities that offered any PhD degrees grew from 89 in 1945 (NSF 2006, Figure 2-1) to 425 in 2003 (NSF 2013, Table 2). Moreover, that increase *understates* the growth of the science and engineering graduate system, as more departments within universities offered the PhD, and existing doctoral programs increased in size. In 1945 American universities produced 936 PhDs in science and engineering. By 2016 U.S. universities graduated 41,342 PhDs, growth by a factor of 44 in a period in which the U.S. population only doubled (NSF 2018a, Table 12). More doctoral programs produced constantly-growing numbers of PhDs, who in turn wanted to get their own research grants and fund their own PhD students, all within a higher educational system that had no mechanisms for limiting the number of PhDs that universities produced.

Analysts often miss this large change in the breadth of the system because they focus on the concentration of federal dollars at the top tier of universities. Federal R&D funds have indeed been concentrated in a small number of U.S. universities, the top twenty of which consists of the usual suspects: the best endowed private universities and a few flagship state universities, especially those with large medical schools (NSF 2018a, Appendix Table 5-6). Only five universities outside the top twenty in 1991 (NSB 1993, Appendix Table 5-4) moved up into the top twenty by 2015. Although the United States has more than 2,000 four-year colleges and universities, 30% of academic R&D spending occurs in those top 20 institutions and about 80% in the top 100, portions that have held steady for decades (NSB 2018a: 5–36 and Figure 5-5).

Despite this concentration, the federal government has managed to spread the greatly-increased wealth. While membership in the top twenty R&D-spending universities has changed very little over time, universities much further down the list now receive enough funding to maintain substantial research activity, dramatically more so than they could have before the war. Universities currently ranked around 100th in federal R&D funding still get more than $100 million from federal agencies (NSF 2018b, Table 5). In addition, researchers at colleges and universities that are not even among the top 100 funding recipients receive enough money to support vibrant research programs in at least a few fields. Ironically, Vannevar Bush’s peer-reviewed project system ended up providing a wide geographic distribution of federal research funds, an outcome he fiercely opposed and that his nemesis Harley Kilgore favored.

American universities were well-primed to take advantage of the increased federal support for R&D and the ensuing imbalance between the supply and demand for the federal R&D funds stemmed in part from institutional features of American higher education, including its rapidly growing size, its high level of fragmentation, the autonomy of individual universities, and the incentives that drive them. No central body has governed all the private and state-supported colleges and universities in the United States. All those institutions have exercised substantial autonomy in making most strategic decisions (Shils 1997: 261–2), such as whether to offer graduate degrees (Ben-David 1974). There are some constraints on this autonomy, usually from governing boards and state legislatures. However, the largest constraint on graduate science programs has always been financial and, prior to the post-war rise of federal R&D spending, there were few opportunities for acquiring such funds.

That rise in federal funding, coupled with the competitive project system of distributing the funds, suddenly created an opening for many universities to try to enter the ranks of research universities. This competitive system fit well with the ideology of the Cold War, which denied the legitimacy of both inherited privilege and central planning (Wolfe 2018) and also fit into an older cultural norm important to the U.S. education system from primary education to universities. Turner (1960: 856) identifies what he calls “contest mobility,” that is, “a system in which elite status is the prize in an open contest and is taken by the aspirants’ own efforts.” Turner focused on individuals and how education institutions could serve them in trying to move up socially, but this cultural norm justifies, even celebrates, the efforts of universities that performed little research prior to the war to compete and acquire some of the material and status rewards from winning research grants.

This growth in research took place during a rapid growth in student enrollments as well. The GI Bill greatly increased university enrollments right after the war (Mettler 2005) and the Baby Boom cohort came of age beginning in the 1960s, pushing enrollments up further. In addition to public policy and demography, the United States experienced a “credential inflation” that spurred more people to seek higher education (Collins [1979] 2019). All of this culminated in enrollments going up from about 1.5 million just before the war to 19.6 million in 2018, after peaking at 21 million in 2010 (U.S. Department of Education). Universities around the country got bigger and became more research-intensive by competing for federal dollars.

Paul Pierson (2004, chap. 1) emphasizes that positive feedback leads to path dependence, which makes institutions and public policies more difficult to change the longer they proceed in the same direction. As more groups and institutions both benefit from and invest in the status quo, they make it more costly to change. As more universities invested in the facilities and faculty they needed to compete successfully for increasing federal R&D funding, the larger and more geographically-spread the constituency for such funding became. Nothing pre-determined this path. Instead, after the critical juncture of World War II, well-placed political elites advocated new institutional arrangements, new structures, rules, and internalized norms, which created new beneficiaries. Vannevar Bush and his allies wanted to greatly increase funding for non-defense R&D while minimizing government control of such research. To operationalize that goal, they promoted the mechanism of competitive funding for individual projects, making federal extramural R&D an instance of institutions that are “*distributional instruments* . . . specifically intended to distribute resources to particular kinds of actors and not to others” (Mahoney and Thelen 2010: 8, emphasis in original). Even if it was not Bush’s intention, these policies greatly expanded the number of those “particular kinds of actors.” The numbers of beneficiaries of the system grew massively, both increasing political support for the system and simultaneously undercutting its ability to function in the long run. Making large changes to that system now looks very risky to its beneficiaries, even as its problems grow. These are the real roots of the financial woes that *The Gathering Storm* and other advocates for science funding decry.

## Long-Term Consequences within the System

The post-Sputnik rates of increase in R&D spending could not last long. In 1963, the science historian Derek de Solla Price pointed out that, before long, if the rapid growth in the R&D system continued, “we should have two scientists for every man, woman, child, and dog in the population, and we should spend on them twice as much money as we had” (Price 1963: 19; see also Weinberg (1963) 1966). Obviously that could not happen, and the first cuts to science funding followed not long after Price’s warning.

But while analysts noticed the problems, they did not always get to their root. By late 1984, the House Science Committee was sufficiently concerned about federal science funding that it commissioned studies and held hearings running to thousands of pages over two years. “An Agenda for a Study of Government Science Policy” identified “Funding Levels” as one of the topics requiring discussion and analysis, but it did not do so with any sense of crisis or impending shortage of funding. Instead, it framed the issue as one of figuring out the “optimal” level of federal funding for science and reviewed the various ways by which the government might determine such a level. These included calculating social benefits or using some percentage of GDP or the total federal budget, among other metrics. Interestingly, the first method for allocating funds the agenda listed was put as the question “Should all good scientists be supported?” (H.R. Rpt 1984: 41–45, quote on 42), which seems naïve now. At committee hearings, witnesses testified to the importance of scientific autonomy, to the linear model of technological innovation as a justification for funding the “best” science, and tied the importance of science to the need for productivity improvements and economic growth. As Harvey Brooks, a prominent science policy expert from Harvard, testified, “Predictions of expected payoff are, I believe, of dubious validity as a basis for allocating investments in science. . . . [more important is] scientific opportunity; that is to say, the prospect of the conceptually significant advances in knowledge independent of potential applications” (H.R. Hearings 1986b: 2).

Despite all the expert testimony that re-affirmed the existing system, other analysts pointed to coming problems. Bruce Smith (1990: 47) pointed out that “While the project system was never the whole of the federal effort, it determined the essential character of the post-war system.” When budgets were increasing rapidly, money could flow both to the well-established and the up-and-coming universities. But at some point demand had to outstrip supply: “the logic of endless growth of R&D budgets has simply collided with the fiscal realities of American politics. Scientists, like any other group, will have to rethink their priorities and show that they are able to accomplish more with fewer resources” (Smith 1990: 168). Smith’s diagnosis of the problem was certainly correct, although he left out one important factor; “scientists” do not, as a group, have the institutional means for “rethinking their priorities” any more than American higher education, as a whole, has the institutional capacity to decide which universities get to create and sustain PhD programs and which do not.

In an effort to address the problem of increasing competition for funds, in 1998 Congress initiated a five-year doubling in current dollars of the funding for NIH, a commitment they completed in 2003 (AAAS 2003) with bipartisan support, raising the budget from $13.1 to $26.4 billion.^5^ Congress achieved that doubling by reducing funding for other fields of science, particularly space and energy R&D, and increasing total non-defense R&D spending overall (AAAS 2003; AAAS 2018). However, even after this budget doubling, success rates for grant applications to NIH soon began declining again. While about one-third of NIH research grant applications were funded in the late 1990s, slightly less than one-fifth have received funding in the last decade (NIH 2018). Similarly, funding success rates at the NSF have declined from 30 to 34% in the early 1990s to 22–24% in the last decade (NSB 2017, Table 1).^6^ In both agencies, the success rates seem to have leveled off, but they could decline further, and researchers in some areas already report anecdotally much lower success rates.

Nonetheless, declining success rates have not led to a drastic change in the American government’s policies for funding science, despite the NAS’s call to arms more than a decade ago (NAS 2007). Both government agencies and universities have muddled along, displacing most of the problems onto scientists themselves, especially those early in their careers. Anecdotal stories about the toll on young scientists have circulated in such high-profile venues as *Science’*s “Working Life” column. In addition, a recent study of three science and engineering disciplines shows that young scientists are dropping out of scientific careers much more rapidly than cohorts that entered those disciplines in the 1960s (Milojevic and Walsh 2018). The National Academies produced a study of post-docs in 2014 that documented their growing numbers and deteriorating situation, with longer times spent in post-docs, salaries that do not keep pace with inflation, and a decreasing chance of getting an academic job when their post-docs were over (IOM 2014, ch. 1–2).

A major source of positive feedback that promotes the persistence of this system is the sheer amount of money the federal government provides to universities. In FY2018, federal funding for higher education R&D expenditures was about $42 billion (NSF 2019, Table 1). In addition, hundreds of universities have invested substantial funds from other sources to compete for the federal funds, deepening the “lock-in” effects (Béland 2010: 574) of federal science policy. That financial commitment and the deeply entrenched norms that drive it create major barriers to disrupting this policy path.

This reluctance to confront the institutional roots of these problems has led science policy elites to frame the problem as an inadequate supply of research money rather than too much demand for it. The 2007 NAS study, *Rising Above the Gathering Storm,* depicted at length the growing problems that beset researchers. They presented numerous policy recommendations, many of them relevant and sensible. But the heart of the matter was money: they argued that the federal government should increase substantially its funding for R&D, especially for the physical sciences and mathematics. Chapter 3 of the report details a long decline after a golden age of funding in the 1960s and 70s: “Federal R&D as a percentage of GDP peaked in the early 1960s and has fallen since then” (NSF 2017: 91). The body of the report avoided stating just how much this increase should be, but a White Paper in Appendix D, which was not part of the official recommendations of the report, stated the numbers baldly: “Increase the budget for mathematics, the physical sciences, and engineering research by 12% a year for the next 7 years within the research accounts of the Department of Energy, the National Science Foundation, the National Institute for Standards and Technology, and the Department of Defense (p. 398).” Such increases would roughly double real spending in seven years, assuming 2% inflation. As appealing as this recommendation might be to scientists, it willfully ignores the constraints of budgetary politics. In addition, would such increases actually increase success rates over the long term or just, as in the case of increases to the NIH budget, bring more proposals out of the woodwork? And what happens after the seven years are up? Federal research programs cannot grow five times faster than the economy forever, as scientists discovered after the 1960s.

*The Gathering Storm* study panel reconvened in 2010 and concluded that things had gotten worse, *Rising Above the Gathering Storm, Revisited: Rapidly Approaching Category 5*. The storm had become a hurricane both because the U.S. government was not spending sufficient sums to implement the Academies’ recommendations and because other countries were. The result was “a system failure” (NAS 2010: 23). While the report acknowledged that there were many demands on the federal budget, its authors insisted that spending for R&D had to be treated differently than other spending because it was so important: “actions such as doubling the research budget are investments that will need to be made if the nation is to maintain the economic strength to provide for its citizens healthcare, social security, national security, and more. One seemingly relevant analogy is that a non-solution to making an over-weight aircraft flight-worthy is to remove an engine” (NAS 2010: 2).

One might add that another non-solution is demanding the repeal of the law of gravity. Calling for ever-greater government funding is ineffectual because it ignores the distributional conflicts that constrain the federal budget. Science advocates often present their claims as a special case, one that legislators and presidents should consider to be above the political fray of competing for budget resources. But no part of the budget can escape budget politics; scientists are competing for funds with dozens of other programs in the context of ever-growing mandatory expenditures, now the largest part of the budget, which bypasses Congressional appropriations committees. The Treasury must simply supply the funds that programs such as Social Security need, based on criteria set out in their authorizing legislation (OMB 2017: 71–72). Congress only gets to decide on the level of spending for the remainder, the defense and non-defense discretionary budget, the latter of which supplies most of the R&D funding that goes to universities. Therefore, the budget politics question is how much of the non-defense discretionary budget goes to R&D. For the last 40 years, the answer has been stable at about 10% (AAAS 2018, R&D as a % of Discretionary Spending). With only small fluctuations, the federal government has funded science at this steady portion of the discretionary budget, regardless of which party controlled Congress or the White House.

But a consistent fraction of the non-defense discretionary budget does not provide enough money to solve the problem of growing induced demand for research funds since, at best, it can only grow at the rate of the overall economy, assuming that federal spending remains a consistent fraction of the GDP and the domestic discretionary budget remains the same fraction of the total federal budget. And that is the optimistic scenario. For more than 50 years total federal spending, mandatory and discretionary, has fluctuated between about 18% and 21% of the GDP, with a bump up to 24.4% during the Obama Administration’s stimulus program (OMB 2018, Historical Table 1.2). Meanwhile, the discretionary budget has shrunk steadily as a percentage of total federal spending, displaced by mandatory spending and interest on the debt. In 1962 the discretionary budget was two-thirds of the total, but had shrunk to about 30% by 2017 (OMB 2018, Historical Table 8.3). These trends constrain the R&D budget even further. To imagine that R&D budgets will continue to shoot up five times faster than economic growth indefinitely, as the NAS proposes, ignores everything we know about federal budgeting. In the long run such increases become arithmetically impossible, but even in the short term they encounter serious political problems.

Nonetheless, one can understand the appeal of these fanciful arguments for constraint-free budgets when considering the political problems that more realistic arguments for increased budgets present for science policy advocates. There are only two ways R&D funding can grow more quickly than the economy, both of them politically sensitive. If advocates for science funding try to claim an ever-larger share of the non-defense discretionary budget, they must argue that funding for R&D is more deserving of support than all the other domestic programs—the grubby business of budget politics. Many of those other programs serve low-income and other disadvantaged communities or supply other important public goods like environmental protection, law enforcement, or workplace health and safety. Do science policy advocates really want to argue for cuts in programs that support desperate short-term needs and often aid the poorest segments of the population?

The alternative route for R&D funding growth is for the federal budget to take a larger share of the economy, which, on its face, is a reasonable idea. Compared to other wealthy countries, the United States has a relatively low percentage of its economy going into the government, even when adding together federal, state, and local taxes. Of the 35 member countries of the Organization for Economic Cooperation and Development (OECD), only Ireland and Mexico have public revenues that are a smaller percentage of their GDPs than the United States (OECD 2017, Table 2.18). Since other prosperous countries have government revenues that take in a much higher percentage of GDP, there is no obvious reason that the United States government could not also do so.^7^ But advocating for such an increase would put science advocates squarely in the middle of one of the oldest ideological conflicts in U.S. history, the argument over the appropriate size of the government’s share of the economy. Taking such a position runs sharply counter to the strategy that they have taken since World War II of arguing that funding for science should be a bipartisan and non-ideological policy issue.

Therefore, if science policy elites want to leave the existing R&D funding system intact, that leaves them with politically unpalatable or substantively ineffective options. They have avoided the unpalatable options of claiming that science funding is more important than low-income housing or that the American federal government should grow from 20% to 30% (or more) of the economy. Instead they have followed an ineffective strategy, arguing for more funding for science as if there were no competition for funds and no ideological disagreement over the proper size of the government, that science is special and should not be subject to the constraints that confront other recipients of federal funds. That strategy has failed to solve the problems science policy confronts precisely because science cannot get an exemption from budgetary and ideological constraints.

## Conclusion: Rethinking Rules, Norms, and Practices

Avoiding these unpalatable and fruitless strategies means seeking larger changes to the R&D funding system. Both the federal government and universities must rethink their institutional norms, rules, structures, and processes that govern the funding of research. Most vexingly, who suffers from the constraints on or changes in the flow of funding? The interactions between the rapidly increasing funding after the war, the competitive project system, and the norms of higher education that prioritize research have led to the unintended result of induced demand for rapid and unceasing growth in research funding, a form of institutional friction (Thelen 1999: 397–400; Lieberman 2002: 697–8). These interactions have unfolded over decades, with problems getting worse only gradually.

For universities, one major factor is the academic norm emphasizing research and publication, which predates World War II, as American academics sought to emulate the German model of the research university, and which found a very congenial home in a few U.S. universities (Ben-David 1974 [1972], ch. 6). After the war, the sharp increases in federal funding led to many more colleges and universities putting a heavy emphasis on research and publications for the hiring and promotion of faculty (Bok 2015: 328–29), a trend that Ben-David (1974 [1972]) noted by the early 1970s. Rapidly increasing post-war research funding made this trend of rising research standards possible, providing scientists at many more universities the opportunity for funding that such research required, which in turn created the positive feedback mechanism that strengthened path dependency. Once funding leveled off, faculty faced intense pressure to maintain their research while having a more difficult time obtaining funding for it.

The extraordinary events of World War II swept away the constraints on federal funding for science, a classic critical juncture, and so created an opening for science policy elites to initiate greatly increased support for science during the war and to argue that future national security would require that the government continue supporting American scientists during peacetime at financial levels unimaginable a few years earlier. The competitive project system opened up that funding to a much larger array of universities than before the war, resulting in a huge expansion of research and doctoral programs. Twelve years after the war, science advocates interpreted the launch of Sputnik as another national crisis, but one that did not challenge the basic institutional rules of science policy, instead calling for even more rapid increases in funding than were already occurring. Those funding increases, in turn, greatly expanded the size of the American academic scientific community, induced greater demand for funding, and deepened universities’ dependence on federal funding and so their defense of the existing system.

The positive feedback loops that reinforce the existing system are still operating; positive feedback can reinforce policies even if they become deeply dysfunctional (Pierson 2004). Moreover, there are no simple criteria to evaluate what would now constitute a functional science policy. So what if senior researchers have to spend increasing amounts of their time writing grant proposals, or junior scientists spend ever more time in post-docs with diminishing prospects of getting permanent positions, or if medical school departments are in ever-more precarious financial situations, or if junior scientists are leaving the profession? The government is still funding science. People at universities are still performing research and publishing the results. Universities are still producing PhDs. The pathologies of that system are getting worse only gradually, making it easier to avoid large-scale institutional change, always a painful process. Instead, leaders of the scientific community bemoan the lack of respect that science gets in American society and make futile appeals for vastly more money. However ineffective that approach may be, it enables science policy elites to avoid directly confronting the institutional problems of science policy, which would enmesh the scientific community in difficult controversies.

An assumption, usually implicit, of most political analyses is that actors in a policy conflict know which policies will advance their interests. However, the problems in science policy derive precisely from the success of the winners, the science policy elites at the end of World War II and their successors, not the machinations of the losers. The winners created a system that avoided geographic or other formulas for distributing funds and instead made funding an open and unconstrained competition, with judgments about the most scientifically deserving projects coming from non-governmental scientists. That competitive project system created the incentives for universities around the country to expand their research portfolios, which in turn led to ever-increasing demands for research funds, the unintended higher-order consequences of this system.^8^ Current science policy elites treat these features of the system as given, a deeply embedded set of norms that few people are willing to question in public. But because the existing policies are not fiscally sustainable, it is, ironically, their supporters, not their opponents, who undermine the current system by avoiding the difficult reforms that lie ahead and so increase the problems in the system.

Universities are already struggling to modify the norms through which they evaluate faculty. For example, as scientific projects become more complex, publications with dozens or even hundreds of authors are becoming more common. How much credit does a faculty member get for being one of those authors, but not the lead author? In addition, colleges and universities have begun creating non-publishing career tracks for faculty, in some cases replacing adjunct faculty who teach single courses for very low pay with full-time faculty who get salaries and benefits and who only teach. These processes have been anything but smooth and present their own possible set of unintended consequences, but universities are gradually changing the composition and functions of their faculty, albeit in an uncoordinated and ad hoc manner.

One of the problems any reforms will confront is the sheer number of science and engineering PhDs the United States produces each year. Should American universities reduce that number? If so, how, and what would be the consequences for the many research projects that depend on PhD students to carry them out? Should the federal government give universities incentives to make such reductions? Or is the problem more cultural than quantitative? Maybe there is a large demand, real or latent, for PhDs outside academia, but universities convey too much disdain about pursuing such career paths by modelling research-oriented faculty positions as the only respectable career for PhDs. That expectation is so deeply embedded in academic culture that one graduate student described telling her advisor about her wish to pursue a non-academic track with the same anxiety and trepidation she felt when she came out about her sexual orientation to her family (Phillips 2018). Sauermann and Roach (2012, Figure 1, p. 3) show that academic jobs are, by far, the most desirable career track for students starting their doctorates in the life sciences and physics, and still popular, though roughly tied with industry and government, for chemistry students. Ginther (2015) elucidates the trends that have led to the decline in tenure track academic jobs in the United States that, coupled with growing numbers of PhDs, put them out of reach for all but a small number of new scientists. By 2015, only 8.1% of those who received their doctorates in the biological sciences in the previous 3–5 years held tenured or tenure-track jobs, down from 17.3% in 1993 (NSB 2018, Table 3–16, p. 3–91).

Federal funding agencies, as well as in universities, will need to enact long-term changes to create a fiscally sustainable R&D system, which includes adopting a different model for how science and engineering research benefits society. The core justification for the existing system, the linear model of innovation, has been thoroughly and effectively critiqued (Narayanamurti and Odumosu 2016, chap. 3; Mowery and Rosenberg 1989, chap. 2; Zachary 1997: 219; Sarewitz 1996: 97–102). To begin, the federal government should rethink the mechanisms it uses to distribute research funding. The existing competitive project system is not the only means for funding quality R&D and may not be the best way to fund science and technology that will have important social benefits. For example, the Department of Agriculture has used a portfolio of methods for distributing research funds, including block grants to universities. The Defense Department’s Advanced Research Projects Agency has used the strong program manager model for funding (H.R. Rep. 1986b). Various OECD countries have used layers of different mechanisms, from project grants to programmatic and institutional support (Paic and Viros 2019: 23–25). While none of them work perfectly, all are worthy of serious analysis as partial replacements for the existing system.

A new system will need to accomplish several goals at once. To be financially sustainable, such a system can grow, but its growth should not exceed the ability of the government to fund it, which over the long term means growing no faster than the economy. Beyond financial sustainability, government funding should provide tangible social benefits. While the government should support some research for its own sake, the classic notion of basic science, there is a serious question about how much money can or should go into that work versus research with a more applied orientation. The NSF has already moved in that direction, requiring since 1997 an assessment of the “Broader Impacts” of all its research project proposals (Frodeman and Holbrook 2011). However, requiring each individual research project to demonstrate its relevance to broad national goals puts the burden on the wrong decision makers, the project principal investigators. If the NSF wants its funding to contribute to such goals, then “[t]he NSF’s capacity to meet broad national goals is best pursued through strategic design and implementation of its programmes, and best assessed at the programme-performance level” (Sarewitz 2011). The individual PI-driven project is more than just a funding mechanism; it is a bottom-up method of setting detailed funding priorities based on proposal pressure from scientists outside of government, which may be quite different from the government’s social goals. In contrast to this standard procedure, the NSF has occasionally engaged in top-down decisions to push the scientific community in particular directions of national need (Sarewitz 2011). Nonetheless, such episodic efforts at pursing national goals leaves most distributional decisions among fields of science subject to past precedent, uncoordinated lobbying, and ad hoc policy decisions rather than any larger strategy.

The project mechanism and the linear model of innovation that underlies it are inextricably bound together in this system, which means that reforming the mechanism to make the system fiscally sustainable requires replacing the model to enable strategic debates for how best to focus federal R&D funds on national needs. A new model will challenge such basic categories as the classic distinction between basic and applied research (Stokes 1997 presents one promising example of an alternative). In addition to social benefits and financial sustainability, a new system should avoid freezing existing privileged positions in place. Scientists and the institutions in which they work need incentives to innovate and maintain quality in their work.

None of this will be easy. Communities are rarely good at making brutal distributional decisions for themselves, and there is no simple alternative that solves these problems. That said, the existing system is clearly unsustainable and in fact is undergoing slow, ad hoc, and painful changes already. Making those changes more deliberate, with mechanisms built in for observation, learning, and modification as new modes of funding begin to function, can save valuable resources and create more realistic expectations on the part of scientists, universities, and governments alike. Those deliberations need to start with a realistic appraisal of the funding streams that the government can provide, and, just as important, with clear recognition of the institutional dysfunction at the heart of the current problems.

## Footnotes

1 Budget data are from Message (various years), Budget Statements “Summary of Expenditures, Classified by Governmental Functions, fiscal year 19xx, compared with prior years,” available from the St. Louis Federal Reserve Bank, https://fraser.stlouisfed.org/. Of the roughly $10 million of “pure” research funding, some fraction of that was performed by government scientists and so there were only a few million available for university-based research.

2 Skilled program managers in funding agencies can influence the peer review process, and occasionally Congress bypasses that process through earmarks for R&D facilities. That said, no other distributional policy puts so much influence in the collective hands of the beneficiaries of the policy.

3 *Encyclopaedia Britannica Online*, s.v. “Nobel Prize,” accessed June 4, 2018, https://www.britannica.com/topic/Nobel-Prize/The-prizes. U.S. winners or winners affiliated with the United States and another country won 249 out of 463.

4 http://www.shanghairanking.com/ARWU2017.html, accessed on June 9, 2018.

5 See the AAAS Historical Dashboard, available at https://www.aaas.org/programs/r-d-budget-and-policy/interactive-dashboards-0.

6 Reports from the 1990s are available in html at https://www.nsf.gov/nsb/publications/pubmeritreview.jsp.

7 Steven Weinberg (2018: 161–3) has explicitly argued for such overall federal budget increases, but he is an exception. Also, as of this writing, the U.S. government has enacted immense disaster relief funding in response to the Covid-19 pandemic. However, those levels of funding are unlikely to continue.

8 I am grateful to an anonymous reviewer for this formulation.
